# Selective inhibition of RET mediated cell proliferation in vitro by the kinase inhibitor SPP86

**DOI:** 10.1186/1471-2407-14-853

**Published:** 2014-11-20

**Authors:** John P Alao, Sona Michlikova, Peter Dinér, Morten Grøtli, Per Sunnerhagen

**Affiliations:** Department of Chemistry and Molecular Biology, University of Gothenburg, Box 462, SE-405 30 Göteborg, Sweden; Department of Chemistry/Organic, KTH Royal Institute of Technology, Teknikringen 30, SE-100 44 Stockholm, Sweden

**Keywords:** RET, FAK, Thyroid cancer, Breast cancer, Estrogen receptor, Kinase inhibitor

## Abstract

**Background:**

The RET tyrosine kinase receptor has emerged as a target in thyroid and endocrine resistant breast cancer. We previously reported the synthesis of kinase inhibitors with potent activity against RET. Herein, we have further investigated the effect of the lead compound SPP86 on RET mediated signaling and proliferation. Based on these observations, we hypothesized that SPP86 may be useful for studying the cellular activity of RET.

**Methods:**

We compared the effects of SPP86 on RET-induced signaling and proliferation in thyroid cancer cell lines expressing RET-PTC1 (TPC1), or the activating mutations BRAF^V600E^ (8505C) and RAS^G13R^ (C643). The effect of SPP86 on RET- induced phosphatidylinositide 3-kinases (PI3K)/Akt and MAPK pathway signaling and cell proliferation in MCF7 breast cancer cells was also investigated.

**Results:**

SPP86 inhibited MAPK signaling and proliferation in RET/PTC1 expressing TPC1 but not 8505C or C643 cells. In TPC1 cells, the inhibition of RET phosphorylation required co-exposure to SPP86 and the focal adhesion kinase (FAK) inhibitor PF573228. In MCF7 cells, SPP86 inhibited RET- induced phosphatidylinositide 3-kinases (PI3K)/Akt and MAPK signaling and estrogen receptorα (ERα) phosphorylation, and inhibited proliferation to a similar degree as tamoxifen. Interestingly, SPP86 and PF573228 inhibited RET/PTC1 and GDNF- RET induced activation of Akt and MAPK signaling to a similar degree.

**Conclusion:**

SPP86 selectively inhibits RET downstream signaling in RET/PTC1 but not BRAF^V600E^ or RAS^G13R^ expressing cells, indicating that downstream kinases were not affected. SPP86 also inhibited RET signaling in MCF7 breast cancer cells. Additionally, RET- FAK crosstalk may play a key role in facilitating PTC1/RET and GDNF- RET induced activation of Akt and MAPK signaling in TPC1 and MCF7 cells.

**Electronic supplementary material:**

The online version of this article (doi:10.1186/1471-2407-14-853) contains supplementary material, which is available to authorized users.

## Background

The REarranged during Transfection (RET) receptor tyrosine kinase (RTK) regulates key aspects of cellular proliferation and survival by regulating the activity of the mitogen- activated protein kinase (MAPK) and PI3K/Akt signaling pathways [1, 2]. RET also interacts directly with other kinases such as the epidermal growth factor receptor (EGFR) and hepatocyte growth factor receptor (MET) and the focal adhesion kinase (FAK) [1, 3, 4]. Deregulated RET activity has been identified as a causative factor in the development, progression and response to therapy of thyroid carcinoma. Elevated RET expression has been associated with the development of endocrine resistance in human breast cancer [5, 6]. A number of studies have also identified RET fusion proteins in lung adenocarcinomas [7–9]. Together, these findings suggest that RET presents an attractive therapeutic target for the treatment of certain cancer subsets.

Despite recent advances, the precise roles of RET in mediating cell proliferation, survival, migration, and resistance to therapy remain unclear. The activity of RTKs and their downstream targets is regulated by a complex array of kinase interactions and feedback loops [10, 11]. Hence, directly targeting RAF kinases can lead to transactivation of RAF dimers, increased activation of MAPK signaling and tumor progression [11, 12]. Further research on the role of RET in regulating these activities is thus important for the development of proper therapeutic strategies. Chemical inhibitors can prove useful for investigating signaling pathways and cell physiology, by complementing other model systems such as those employing protein over-expression, chemical- induced dimerization (CID) and siRNA technology [13, 14]. For instance, signaling events often occur in the range of seconds and the ability to rapidly inhibit signaling can be extremely useful for investigations of this nature. Studies on structure- activity relationships using cell line models can also provide insights that direct the design and synthesis of novel kinase inhibitors. Unfortunately, the usefulness of kinase inhibitors in particular, is limited by their relative lack of selectivity. It can thus be difficult to specifically link observed cellular responses to inhibition of the desired target protein. Furthermore, the off target effects of kinase inhibitors can result in undesirable side effects if and when they are employed clinically [11, 15, 16]. Several kinase inhibitors with differential selectivity towards RET have been reported to date. Almost without exception, these inhibitors target several other kinases apart from RET with equal or higher affinity and accordingly induce a diverse range of effects in different cell lines (Table 1) [17–19]. Several of these compounds have entered clinical trials with promising results [1]. While multi-kinase inhibition might be beneficial for cancer treatments, it is also associated with a higher incidence of side effects. The inhibition of vascular endothelial growth factor receptor 2 (VEGFR2), in particular, has been associated with undesirable side effects [17]. The inhibition of multiple kinases by an inhibitor can severely restrict its usefulness as a chemical tool [13, 20, 21]. For instance, RET has been shown to functionally interact with several other kinases such as EGFR, FAK, and MET [3, 4, 22–24]. Furthermore, BRAF and p38MAPK are downstream targets of RET [5]. Kinase inhibitors that simultaneously inhibit RET and its downstream targets (or kinases it interacts with) will produce results in cell based assays that are difficult to interpret [13, 20, 21]. The continued design and synthesis of novel inhibitors with selective activity towards RET is thus important [17, 18, 25].

**Table 1:** **Kinase inhibitors with inhibitory activity towards RET**

Inhibitor	Targets (IC _50_)	Reference
PP1	Lyc (5nM), fyn (6 nM), Src (170 nM), Csk (520 nM), CK1δ (1060 nM), p38MAPK (640 nM), RET (80 nM)	[20, 26, 27]
RPI-1	MET (7.5 μM), RET (170 nM),	[28–30]
PHA-739358 (Danusertib)	Aurora kinase A/B/C (13 nM/79 nM/61 nM), BCR-ABL (25 nM), RET(31 nM), FGFR1 (47 nM)	[31]
TG101209	JAK2 (6 nM), FLT3 (25 nM), RET (17 nM)	[32]
SU 5416 (Semaxanib)	RET (944 nM), VEGFR (nM), KIT, MET, FLT3	[33, 34]
SU11248 (Sunitinib)	RET (224 nM), VEGFR2 (4 nM), FLT3 (8–14 nM), KIT (1–10 nM), PDGFRβ (39 nM), CSF1R (50–100 nM)	[35]
XL184 (Cabozantinib)	VEGFR2 (0.035 nM), MET (1.3 nM), RET (4 nM), KIT (4.6 nM), FLT-1/3/4 (12 nM/ 11.3 nM /6 nM, 14.3 nM), TIE2 (14.3 nM), AXL (7 nM)	[36]
BAY 43–9006 (Sorafenib)	RET (5.9- 47 nM), BRAF (25 nM), VEGFR1/2/3 (20–90 nM), FLT3 (33 nM), p38MAPK (38 nM), PDGFRβ (57 nM), KIT (68 nM)	[35, 37]
ZD6474 (Vandetanib)	RET (130 nM), VEGFR2 (40 nM), VEGFR3 (110 nM), EGFR (500 nM)	[38, 39]
AP24534 (Ponatinib)	RET (7 nM), ABL (0.4 nM), Lyn (0.2 nM), FLT3 (13 nM), KIT (13 nM), FGFR1 (2 nM), PDGFRα (1 nM), Src (5.4 nM), VEGFR2 (2 nM)	[40, 41]
NVP-AST487	RET (880 nM), KDR (170 nM), FLT-4 (790 nM), KIT (500 nM), FLT-3 (520 nM), ABL (20 nM)	[42]
NVP-BBT594	RET (~100 nM), JAK2 (1 nM), Tyk2 (1 nM), JAK3 (5 nM), JAK1 (15 nM), FAK (100 nM), IRK-3P (200 nM), ZAP70 (200 nM), FGFR2 (940 nM)	[43, 44]

We recently reported the design and synthesis of a small library of selective, cell permeable kinase inhibitors with activity against RET [45]. The lead compound (SPP86) [45] has previously been shown by us to exhibit high selectivity towards RET and potently inhibits its activity *in vitro*. Although SPP86 shows high selectivity for RET *in vitro*, it also inhibited EPHA1, FGFR1, FGFR2, FLT4, LCK, YES at low doses (<0.4 μM) under these conditions. As such, its selectivity profile differs from that of other kinase inhibitors reported to inhibit RET activity. Furthermore SPP86 is cell permeable and inhibits RET signaling in human cancer cell lines at low concentrations [45]. Our observations suggest that SPP86 may be a useful chemical tool for studies on RET signaling in cancer models. In this study, we further investigated the utility of SPP86 as a chemical tool for studies on RET signaling in human cancer cell lines. Based on its selectivity profile, we predicted that low doses of SPP86 would exert little or no effect on the signaling and proliferation of cell lines that do not depend on RET for these activities. We compared the effect of SPP86 on MAPK kinase signaling and proliferation in RET/PTC1 (TPC1), BRAF^V600E^ (8505C) and RAS^G13R^ (C643) expressing thyroid cancer cell lines. Widening the scope beyond cancer types traditionally considered to be RET-driven, we also investigated the effect of SPP86 on RET- induced ERα phosphorylation and proliferation in MCF7 breast cancer cells.

## Methods

### Reagents

The RET inhibitor SPP86 was synthesized by a literature procedure [45]. Stock solutions of SPP86 (10 mM) in DMSO were stored at 4°C and diluted just prior to use. 17-β estradiol (E2), 4- hydroxy tamoxifen (4-OHT) and insulin were obtained from Sigma-Aldrich (Stockholm, Sweden) dissolved in ethanol and stored at 4°C. PF-573228 was from Tocris Bioscience (Bristol, United Kingdom), dissolved in DMSO and stored at -20°C. ICI182,780 was from Tocris Bioscience dissolved in ethanol and stored at -20°C. Sorafenib (BAY43-9006) was obtained from AH Diagnostics AB (Skärholmen, Sweden) and stock solutions in DMSO were stored at -20°C. Recombinant human GDNF was obtained from R&D systems (Abingdon, United Kingdom) and was reconstituted and stored according to the supplier’s instructions.

### Cell culture

MCF7 breast cancer cells from in-house stocks were maintained in Dulbecco’s modified eagle medium (DMEM) supplemented with heat inactivated 10% (v/v) fetal bovine serum (FBS), 2 mM L-glutamine, 100 units/ml penicillin and 100 μg/ml streptomycin at 37°C in humidified 5% CO_2_. 8505C, C643 and TPC1 thyroid cancer cell lines were maintained in RPMI 1640 under similar conditions. The 8505C and C643 cells as well as the TPC1 cells were kind gifts from P. Soares and L. Mologni respectively. For estrogen and serum deprivation, MCF7 cells were cultured for 3 days in phenol red free RPMI 1640 supplemented with 10% (v/v) charcoal stripped FBS followed by 24 h in the same medium containing 1% (v/v) charcoal stripped FBS. For 4-OHT response assays, MCF7 cells were cultured for 3 days in phenol red free RPMI 1640 supplemented with 10% (v/v) charcoal stripped FBS followed by 24 h in the same medium containing 0.1% (v/v) charcoal stripped FBS.

### Antibodies

Antibodies directed against RET (C31B4), Akt1 (2H10), phospho-Ser473 Akt (193H12), p70 S6 Kinase, phospho-Thr389 p70 S6 Kinase (108D2), p44/42 MAPK (ERK1/2) (137 F5), phospho-Thr202/Tyr204 p44/42 MAPK (ERK1/2) (197G2), phospho- Src Tyr416, (D49G4), Src (36D10) and phospho-Ser167 (D1A3) ERα were from Cell Signaling Technologies (Bionordika (Sweden) AB, Stockholm, Sweden). Antibodies directed against β- catenin (B-9), phospho- Tyr654 β- catenin (1B11), cyclin D1 (DCS-6), PARP-1/2 (H-250), RET (C-19), phospho-Tyr1062 RET, PARP (H-250) and Sp1 (E3) were from Santa Cruz Biotechnology (Heidelberg, Germany) and against ERα (6 F11) from Leica Microsystems AB (Kista, Sweden). Antibodies directed against phospho- Tyr576 FAK and FAK were from Invitrogen (Lidingö, Sweden). Monoclonal antibodies directed against actin and α-tubulin were from Sigma-Aldrich.

### Cell viability assays

For cell viability assays, cells were seeded in 96-well plates at optimal cell density to ensure exponential growth for the duration of the assay. After 24 h preincubation, growth medium was replaced with experimental medium containing the appropriate drug concentrations or vehicle controls (0.1% or 1.0% v/v DMSO). After 48 h incubation, cell viability was measured using PrestoBlue™ Cell Viability Reagent (Invitrogen) according to the manufacturer’s instructions. Fluorescence was measured at the excitation and emission peaks for resorufin (544 and 590 nm respectively). Results were expressed as the mean ± S.E. for six replicates as a percentage of vehicle control (taken as 100%). Experiments were performed independently at least three times. Statistical analyses were performed using a two tailed Student’s *t* test. *P* <0.05 was considered to be statistically significant.

### Immunoblotting

Cells treated as indicated were washed with ice-cold phosphate buffered saline (PBS) and lysed directly in ice-cold HEPES buffer [50 mM HEPES (pH 7.5), 10 mM NaCl, 5 mM MgCl_2_, 1 mM EDTA, 10% (v/v) glycerol, 1% (v/v) Triton X-100 and a cocktail of protease inhibitors (Roche Diagnostics Scandinavia AB, Bromma, Sweden)] at 4°C for 30 min with gentle agitation. The supernatants were either analyzed immediately or stored at -80°C. Equivalent amounts of protein (20 – 50 μg) from total cell lysates were resolved by SDS-PAGE and transferred onto ‘nitrocellulose membranes. Membranes were blocked in blocking buffer [5% (w/v) nonfat dried milk, 150 mM NaCl, 10 mM Tris (pH 8.0) and 0.05% (v/v) Tween 20]. Proteins were detected by incubation with primary antibodies at appropriate dilutions in blocking buffer overnight at 4°C. Blots were then incubated at room temperature with horseradish peroxidase-conjugated secondary antibody. Bands were visualized by enhanced chemiluminescence (Supersignal West Pico; Pierce, Nordic Biolabs AB, Täby, Sweden) followed by exposure to autoradiography film (General Electric Bio-Sciences, Uppsala, Sweden). Antibodies directed against PARP [46] or tubulin were used to monitor gel loading. Cytoplasmic and nuclear extracts were prepared using an NE-PER extraction kit (Thermo Scientific Inc., Rockford, IL, USA) according to the manufacturer’s instructions.

### Immunofluorescence microscopy

Cells were grown on sterile glass coverslips in 6-well plates to 80% confluence in media before being washed three times in PBS. Cells were fixed in 4% formaldehyde/PBS at room temperature for 10 minutes. Coverslips were washed twice in PBS and permeabilized in 0.2% Triton X100/PBS for 15 minutes. Following another three washes in PBS, coverslips were blocked in 3% bovine serum albumin (BSA)/PBS at room temperature for 30 min. Monoclonal antibodies to β- catenin (B-9) were applied in 3% BSA/PBS overnight. Cells were then washed 3 times in PBS, and incubated with a fluorescein isothiocyanate (FITC) -conjugated bovine or goat anti-mouse secondary antibody (1:200) (Santa Cruz Biotechnology) at room temperature for 1 h. After a final three washes, coverslips were mounted on glass slides with Vectorshield containing 4′,6′-diamidino-2-phenylindole (DAPI) (Vector Laboratories Ltd., Peterborough, United Kingdom). Alternatively, cells were stained with FITC- conjugated phalloidin. Images were obtained with a Zeiss AxioCam on a Zeiss Axioplan 2 microscope with a 100 × objective using the appropriate filter sets.

## Results

We investigated the effect of SPP86 on ERK1/2 phosphorylation in thyroid cancer derived cell lines expressing the RET/PTC1 rearrangement (TPC1), BRAF^V600E^ (8505C) or RAS^G13R^ (C643) mutations [47, 48]. These mutations have previously been shown to induce constitutive activation of the MAPK signaling pathway in these cell lines [47–49]. Since TPC1 but not 8505C and C643 cells depend predominantly on RET/PTC1 signaling for proliferation, we hypothesized that SPP86 should only inhibit the proliferation of the former. Sorafenib, which inhibits both RET and RAF family kinases, was used as an internal control in these experiments.

### SPP86 inhibits MAPK pathway activation in RET/PTC1 expressing cell lines

**Figure 1:**
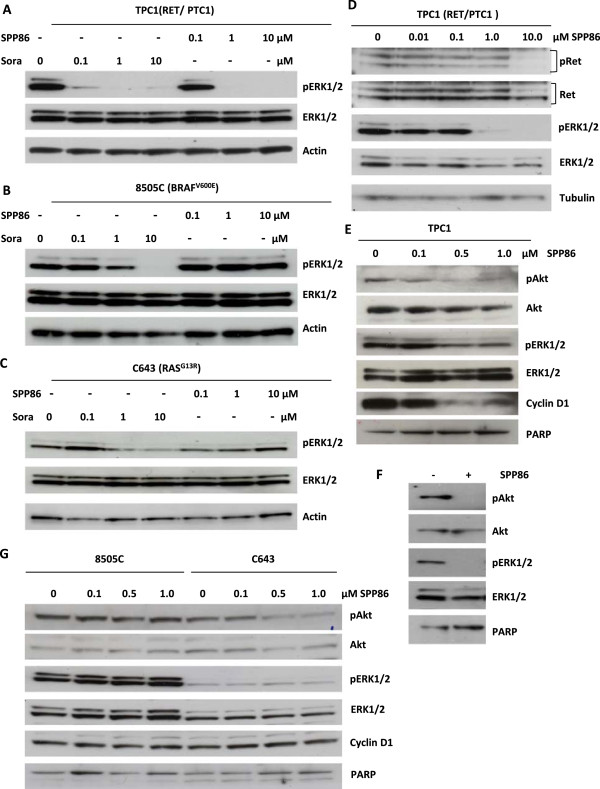
**SPP86 selectively inhibits RET- induced ERK1/2 phosphorylation in thyroid cancer cell lines. (A)** TPC1 cells were cultured overnight in media containing 0.1% FBS and exposed to the indicated concentrations of sorafenib or SPP86 for 90 min in similar media. Total lysates were resolved by SDS-PAGE and probed with antibodies directed against phosphorylated (Thr202/Tyr204) and total ERK1/2. Monoclonal antibodies directed against actin were used to monitor gel loading. **(B)** 8505C cells expressing mutant BRAF^V600E^ and **(C)** C643 expressing mutant RAS^G13R^ were treated as in A. **(D)** TPC1 cells were treated as in C. Total lysates were resolved by SDS-PAGE and probed with antibodies directed against phosphorylated (Tyr1062), total RET, phosphorylated ERK1/2 (Thr202/Tyr204) and total ERK1/2. Monoclonal antibodies directed against tubulin were used to monitor gel loading. **(E)** TPC1 cells were cultured in media containing 0.1% FBS overnight and then exposed to the indicated concentrations of SPP86 for 20 h under the same conditions. Total lysates were resolved by SDS- PAGE and membranes were probed with the indicated antibodies. Monoclonal antibodies directed against PARP were used to monitor gel loading. **(F)** TPC1 cells cultured in media containing 0.1% FBS were exposed to the indicated concentration of SPP86 for 90 min. Total cell lysates were resolved by SDS- PAGE and probed with antibodies directed against phosphorylated and total RET or phosphorylated and total ERK1/2. PARP was used instead of actin or tubulin to monitor gel loading, to enable the simultaneous detection of Akt and ERK1/2 on the membrane. **(G)** 8505C and C643 cells were cultured overnight in media containing 0.1% FBS. Cells were exposed to the indicated concentrations of SPP86 for 20 h. Lysates were probed with antibodies directed against the indicated proteins.

As previously reported [45], SPP86 effectively inhibits ERK1/2 phosphorylation in TPC1 cells expressing the RET/PTC1 rearrangement at a concentration of 1 μM (Figure 1A). In contrast, SPP86 had no effect on ERK1/2 phosphorylation in 8505C or C643 cells (Figure 1B and C). Sorafenib, which targets both RET and RAF kinases, effectively inhibited ERK1/2 phosphorylation in TPC1 cells at a concentration of 0.1 μM (Figure 1A). Sorafenib (10 μM) also inhibited ERK1/2 phosphorylation in 8505C cells, and to a lesser extent in C643 cells consistent with previous reports [49] (Figure 1B and C). The differential sensitivity of 8505C and C643 cells to SPP86 and sorafenib likely results from the latter’s effect on RAF signaling [49]. Interestingly, SPP86 induced only modest inhibition of RET/PTC1 phosphorylation Tyr1062 at a concentration of 1 μM (Figure 1D). Complete inhibition required 10 μM of SPP86 (Figure 1D). Thus, the ability of SPP86 to abolish ERK1/2 phosphorylation at 1 μM does not strictly correlate with its inhibition of RET phosphorylation. Similar observations have previously been reported with the RET inhibitor RPI-1 [28]. SPP86 also inhibited Akt phosphorylation on Ser473 at a concentration of 1.0 μM (Figure 1E and F). Prolonged exposure (20 h) to 0.5- 1 μM SPP86 was also associated with a decline in cyclin D1 levels in this cell line (Figure 1E). In contrast, prolonged exposure to SPP86 did not affect ERK1/2 phosphorylation or cyclin D1 expression in 8505C and C643 cells (Figure 1G). We noted however, that prolonged exposure to SPP86 (0.5- 1.0 μM) was associated with a decrease in Akt Ser473 phosphorylation in C643 in cells (Figure 1G). C643 cells express wild type RET [47]. SPP86 may thus inhibit RET- mediated Akt activation in this cell line. These results demonstrate that unlike sorafenib, SPP86 appears to selectively inhibit RET/PTC1- activated MAPK signaling in these cell lines.

**Figure 2:**
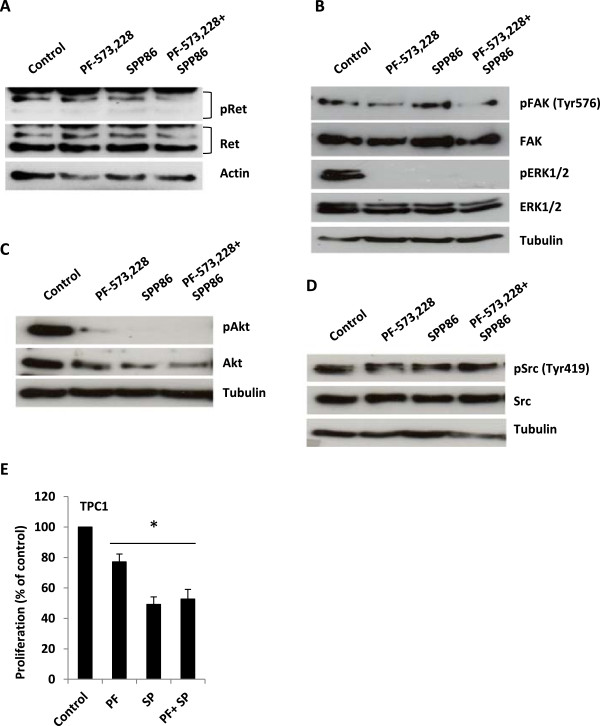
**SPP86- mediated RET inhibition does not abolish its phosphorylation TPC1 cells. (A)** TPC1 cells were cultured overnight in media containing 0.1% FBS and exposed to 2.5 μM PF573228 and/or 1 μM SPP86 for 90 min in similar media. Total lysates were resolved by SDS-PAGE and probed with antibodies directed against phosphorylated (Tyr1062) and total RET. Actin was used to monitor gel loading. **(B- D)** Total lysates were resolved by SDS-PAGE and probed with antibodies directed against the indicated proteins. Tubulin was used to monitor gel loading. **(E)** TPCI cells were grown in media containing 0.1% FBS and the left untreated or cultured in the presence of 2.5 μM PF573228 and/or 1 μM SPP86 for 72 h. Viability was expressed as a percentage of the untreated control population. The data in each panel represent the mean of 3 experiments ± S. E.; *p <0.01 treated *vs.* untreated for each series.

We next investigated if FAK could maintain RET phosphorylation on Tyr1062 despite the inhibition of RET autophosphorylation by SPP86 [24]. Exposure to 2.5 μM of the FAK inhibitor PF573228 [50] alone, did not inhibit RET phosphorylation in TPC1 cells (Figure 2A). In contrast, exposure to 2.5 μM of the FAK inhibitor PF573228 in combination with 1 μM SPP86 was sufficient to inhibit RET phosphorylation on Tyr1062 (Figure 2A). Interestingly, PF273228 and SPP86 inhibited Akt and ERK1/2 phosphorylation to a similar degree (Figure 2B and C). Exposure to PF573288 but not SPP86 was associated with a reduction of FAK Tyr576 phosphorylation (Figure 2B). Neither PF273228 nor SPP86 suppressed Src phosphorylation (Figure 2D). SPP86 inhibited TPC1 cell proliferation more effectively than PF573228 (49% proliferation *vs.* 77%) but no additive effect was observed following co-exposure to both drugs (Figure 2E).

**Figure 3:**
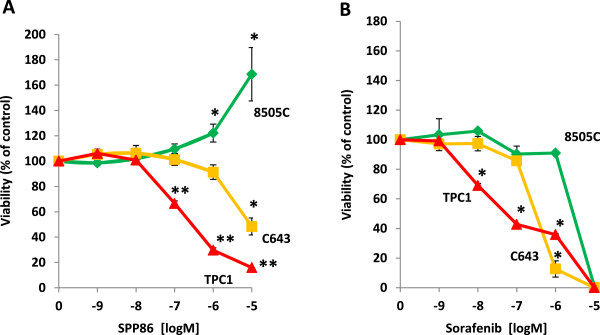
**SPP86 selectively inhibits RET- mediated proliferation in thyroid cancer cell lines. (A)** TPC1 cells expressing RET/PTC1, 8505C cells expressing mutant BRAF^V600E^ and C643 expressing mutant RAS^G13R^ were cultured in medium containing 0.1% FBS in the presence of increasing doses of sorafenib or SPP86 for 72 h. Viability was expressed as a percentage of the untreated control population. The data in each panel represent the mean of at least 3 experiments ± S. E.; *p <0.05 for 8505C cells at 1–10 μM and C643 cells at 10 μM SPP86 *vs.* control, **p <0.0001 for TPC1 cells at 0.1- 10 μM SPP86 *vs*. control. **(B)** TPC1, 8505C and C643 cells were exposed to increasing concentrations of sorafenib in media containing 0.1% FBS for 72 h. Viability was expressed as a percentage of the untreated control population. The data in each panel represent the mean of 3 experiments ± S. E. for C643 and 8505C cells and the mean of 2 experiments ± S. E. for TCP1 cells; *p <0.05 for 0.1- 1.0 μM for TCP1 cells, 1.0 μM for C643 cells.

Our observations strongly suggested that SPP86 would selectively inhibit RET- induced proliferation. We thus compared the effect of sorafenib and SPP86 on the proliferation of TPC1, 8505C and C643 cells in media containing 0.1% serum (i.e. low culture conditions). Under these conditions, signaling pathway activation is predominantly under the control of the respective oncogenes expressed by these cell lines. C643 and TPC1 cells showed marked differential sensitivity to SPP86 (IC_50_, 61.5 vs 1.5 μM for C643 and TPC1 cells respectively) (Figure 3A). In contrast, 8505C cells grew poorly under low serum conditions (data not shown), but their proliferation was enhanced when exposed to doses of SPP86 from 1–10 μM (Figure 3A). Under similar conditions, the IC_50_ values for sorafenib were 3.1 μM, 0.28 μM, and 33.3 μM for C643, TPC1 and 8505C cells respectively (Figure 3B). In general, low doses of SPP86 do not inhibit the activity of signaling proteins downstream of RET or other kinases that directly interact with it [45]. SPP86 thus selectively inhibited the proliferation of TPC1 cells dependent on oncogenic RET but not 8505C and C643 cells respectively dependent on oncogenic BRAF and RAS.

### SPP86 inhibits RET signaling in ERα positive breast cancer cells

**Figure 4:**
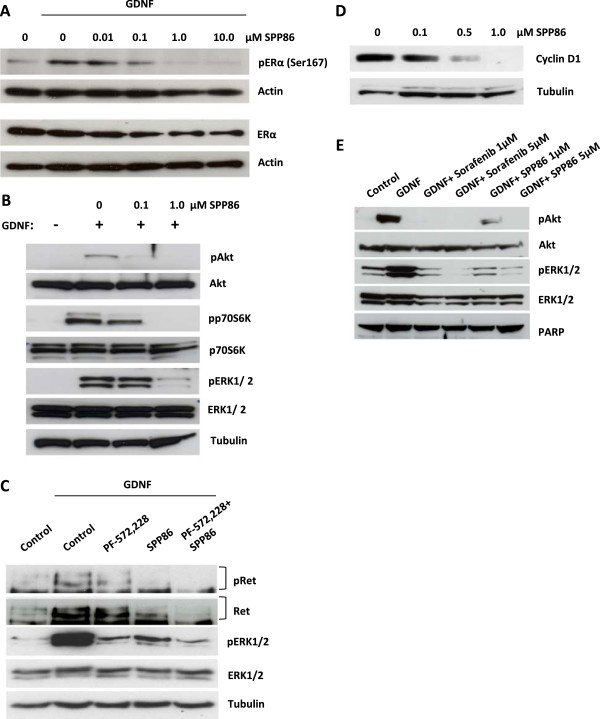
**SPP86 inhibits RET- induced ERα** **phosphorylation and proliferation in MCF7 cells. (A)** Estrogen- deprived and serum starved MCF7 cells were exposed to the indicated concentrations of SPP86 for 30 min. The cells were then exposed to 10 ng/ml GDNF for another 30 min. Total lysates were resolved by SDS-PAGE and probed with antibodies directed against phospho- Ser167 and total ERα. Actin was used as a loading control. **(B)** Estrogen- deprived and serum starved MCF7 cells were pretreated with the indicated concentrations of SPP86 for 30 min and then exposed to 10 ng/ml of GDNF for a further 45 min. Total lysates were probed with the indicated antibodies. **(C)** MCF7 cells were grown in media growing 1.0% FBS overnight, pretreated with 2.5 μM PF573228 and/or 1 μM SPP86 for 40 min and then exposed to 10 ng/ml of GDNF for a further 20 min in similar media. Total lysates were resolved by SDS-PAGE and probed with antibodies directed against phosphorylated (Tyr1062), total RET, phosphorylated ERK1/2 (Thr202/Tyr204) and total ERK1/2. Antibodies directed against tubulin were used to monitor gel loading. **(D)** MCF7 breast cancer cells were cultured in media containing 1% FBS and then exposed to the indicated doses of SPP86 for 24 h. Total lysates were probed with antibodies directed against cyclin D1 and tubulin. **(E)** Estrogen- deprived and serum starved MCF7 cells were pretreated with the indicated concentrations of sorafenib or SPP86 and treated as in C. Antibodies directed against PARP were used to monitor gel loading.

In addition to its role in thyroid cancers, RET has been shown to functionally interact with ERα in human breast cancer cell lines [5, 6, 51, 52]. We thus evaluated the utility of using SPP86 to interrogate RET signaling in MCF7 breast cancer cells. Firstly, we studied the effect of SPP86 on RET- mediated ERα phosphorylation on serine residue 167 (Ser167). Estrogen deprived and serum starved MCF7 cells were exposed to 10 ng/ml GDNF in the absence or presence of increasing does of SPP86. In these experiments, SPP86 effectively inhibited GDNF/RET- induced ERα phosphorylation at a concentration of 0.1 μM (Figure 4A). Higher concentrations of SPP86, 1–10 μM, reduced ERα phosphorylation even below baseline levels. In addition, exposure of MCF7 cells to SPP86 was associated with a moderate decrease in ERα levels in these experiments (Figure 4A) We next investigated the effect of SPP86 on RET- mediated activation of these pathways in MCF7 cells. SPP86 inhibited GDNF/ RET- induced phosphorylation of Akt and its downstream signaling at concentrations as low as 0.1 μM (Figure 4B). We noted that SPP86 inhibited phosphorylation of Akt more effectively than that of its downstream target p70S6K at this concentration. Similarly, SPP86 inhibited Akt phosphorylation at markedly lower concentrations than those required to inhibit MAPK phosphorylation (0.1 vs. 1.0 μM) (Figure 4B). SPP86 effectively inhibited GDNF- induced RET phosphorylation Tyr1062 at a concentration of 1.0 μM. In contrast, FAK inhibition with PF573228 only moderately inhibited RET phosphorylation. Co- exposure to PF573228 and SPP86 however, exerted an additive inhibitory effect on RET phosphorylation (Figure 4C). Both PF573228 and SPP86 inhibited GDNF- induced ERK1/2 and Akt phosphorylation (Figure 4C and Additional file 1A). Prolonged exposure of MCF7 cells to SPP86 also lead to the suppression of cyclin D1 expression (Figure 4D). We next compared the effects of sorafenib and SPP86 on PI3K/Akt and MAPK pathway signaling, with a view to discriminate the direct effects of RET inhibition from those of a combined inhibition of RET and RAF. In these experiments, estrogen deprived and serum starved MCF7 cells were exposed to 10 ng/ml GDNF alone or in the presence of either sorafenib or SPP86. Analyses of the relative levels of phosphorylated Akt and ERK1/2 demonstrated that both compounds effectively block GDNF- induced RET signaling at concentrations as low as 1 μM (Figure 4E). We noted however, that sorafenib inhibited Akt and ERK1/2 slightly more effectively than SPP86 under these conditions (Figure 4E). These differential effects on PI3K/Akt and MAPK signaling may result may stem from the fact that sorafenib and SPP86 target different kinases at low concentrations. The enhanced inhibition of MAPK signaling observed with sorafenib may also result from the fact that it targets both RET and RAF family kinases [37, 45].

**Figure 5:**
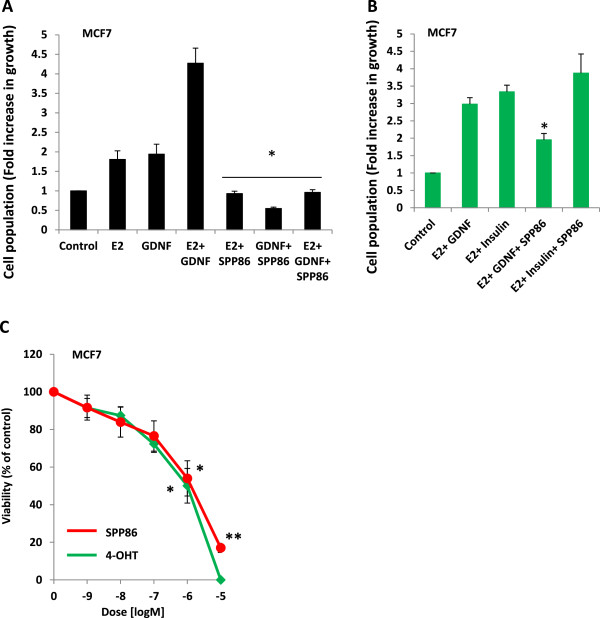
**SPP86 inhibits RET- mediated proliferation. (A)** Estrogen- deprived and serum starved MCF7 cells were left untreated or exposed to 1 ng/ml E2 and/or 10 ng/ml GDNF alone or in combination with 1 μg/ml SPP86 in phenol red- free media for 7 days. Proliferation was expressed as fold increase in growth relative the untreated control population. The data represent the mean of 3 experiments ± S. E; *p <0.05 treated *vs.* untreated for each series. **(B)** Estrogen- deprived and serum starved MCF7 cells were cultured in the presence of 1 nM E2 (estrogen) together with 10 ng/ml of GDNF or 10 ng/ml insulin in the presence or absence of 1 μM SPP86 for 72 h. Proliferation was expressed as fold increase in growth relative the untreated control population. The data represent the mean of 3 experiments ± S. E; *p <0.05. **(C)** Estrogen- deprived and serum starved MCF7 cells exposed to increasing concentrations of SPP86 or 4-OHT in phenol red- free media containing 1 ng/ml E2 and 10 ng/ml GDNF for 7 days. Viability was expressed as a percentage of the untreated control population. The data in each panel represent the mean of 3 experiments ± S. E.; *p <0.05, **p <0.0001.

Since these observations suggested that SPP86 disrupts ERα- RET crosstalk, we investigated the effect of SPP86 on the proliferation of MCF7 cells. Estrogen deprived and serum starved cells were cultured in the presence of 1 ng/ml β- estradiol (E2) or 10 ng/ml GDNF alone and in combination in the presence of 1 μM SPP86 for 7 days. SPP86 effectively inhibited E2 and/or GDNF- induced proliferation (*p* <0.05) (Figure 5A). In contrast, SPP86 did not inhibit proliferation when MCF7 cells were co-exposed to 1 ng/ml E2 and 5 ng/insulin under similar conditions (Figure 5B). We next compared the effect of SPP86 and tamoxifen on the proliferation of MCF7 cells. Estrogen deprived and serum starved cells were cultured in the presence of 1 ng/ml β- estradiol (E2) and 10 ng/ml GDNF with increasing doses of either SPP86 or tamoxifen, in medium containing 1 ng/ml β- estradiol (E2) and 10 ng/ml GDNF and incubated for 7 days. In these experiments, SPP86 and tamoxifen inhibited proliferation to a similar degree with IC_50_ values of 1.0 and 1.4 μM respectively (Figure 5C).

## Discussion

We have investigated the effect of SPP86, a novel small molecule kinase inhibitor with selective activity towards RET on cancer cell proliferation. SPP86 is cell permeable, potently inhibits RET activity *in vitro* and *in vivo*, and exhibits a unique selectivity profile that differs from previously reported inhibitors with activity towards this kinase [45]. Deregulated RET activity has been associated with the development, progression and/or resistance to therapy of certain thyroid, breast and lung cancer subtypes. Together, these studies have identified RET as a potentially important therapeutic target in these subtypes of thyroid, breast and lung cancers [5–9]. Further studies on RET will be required however, if effective treatment regimens that target this kinase are to be developed. Due to the rapidity of their actions, small molecule tyrosine kinase inhibitors have been extremely useful as chemical tools to study the physiological roles of the pathways regulated by these enzymes [13, 14, 21]. Most if not all kinase inhibitors target more than one kinase, leading to potentially confounding or erroneous results when used to study cellular physiology. Several small molecules with inhibitory activity towards RET have been reported (Table 1). This problem can be partially circumvented; by using two or more RET inhibitors of dissimilar structure for studies of this nature [13, 20, 21]. Given that no purely selective inhibitors of RET exist, the continued characterization of small molecules that target RET is desirable.

The differential selectivity profile of SPP86 suggested it might be useful for studies on the cellular functions of RET [45]. As previously reported for other RET inhibitors [17–19, 45], SPP86 inhibits RET mediated activation of the PI3K/Akt and MAPK pathways at low doses (0.1- 1 μM) in a cell line expressing oncogenic RET. In this study, we have demonstrated that SPP86 selectively inhibits this activity in a thyroid cancer cell line expressing RET/PTC1 but not in others with activating mutations in BRAF (V600E) or Ras (G13R) which lie downstream of RET. Furthermore, SPP86 selectively inhibited the proliferation of the former at similar concentrations while having little or no anti-proliferative effect on the latter. Interestingly, SPP86 appears to enhance the proliferation of 8505C cells which express constitutively activated BRAF^V600E^ under low serum conditions. It remains to be determined, if this effect resulted directly from the SPP86 mediated inhibition of RET.

Surprisingly, we observed only partial suppression of RET phosphorylation on Tyr1062 following exposure to low doses of SPP86. The near complete inhibition of Akt and ERK1/2 by SPP86 at these doses, did not correlate with inhibitory effects on RET phosphorylation. While similar observations have been previously reported [28], the reasons for this discrepancy remain unclear. FAK has been shown to phosphorylate RET on Tyr1062 [24]. In our studies, FAK inhibition by PF573228 did affect RET phosphorylation in TPC1 cells. Co-exposure to PF273228 and SPP86 however, clearly inhibited RET phosphorylation. SPP86 did not inhibit the activity of FAK or its activating kinase Src in our studies. It is possible, that FAK maintains the phosphorylation of RET on Tyr1062 despite the inhibition of the latter’s autophosphorylation by SPP86. Interestingly, exposure to PF573228 or SPP86 inhibited Akt and ERK1/2 to a similar degree in TPC1 cells. In contrast, low concentrations of SPP86 clearly inhibited GDNF- induced RET autophosphorylation in MCF7 cells. Exposure to PF573228 alone only marginally inhibited GDNF- induced RET autophosphorylation but enhanced the inhibitory effect of SPP86 on this activity. PF573228 also inhibited RET- dependent activation of Akt and ERK1/2 in MCF7 cells to a similar degree as SPP86 (Figures 2 and 4). The precise role of FAK in regulating Akt and ERK1/2 phosphorylation as well as proliferation in MCF7 and TPC1 cells will require further studies. Our findings suggest however, that RET autophosphorylation is insufficient to activate downstream signaling and requires FAK activity. TPC1 cells have also been shown to express FGFR1, a target of SPP86 [53]. The role of FGFR1 in regulating the aforementioned effects on cell signaling in TPC1 cells has not been reported. Therefore we cannot rule out at present, that the inhibitory effect of SPP86 on TPC1 cell proliferation partially results from the inhibition of FGFR1. RET/PTC1 is the main oncogenic driver in the TPC1 cell line and activates both the Akt and MAPK signaling pathways [54, 55]. The observed selective effect on RET driven proliferation therefore suggests that SPP86 predominantly inhibits signaling via this RTK.

The effectiveness of endocrine therapy for ERα positive breast cancer is limited by the development of resistance. Increased RTK signaling leads to estrogen independent ERα activation and resistance to tamoxifen and aromatase inhibitors [56]. RET interacts functionally with ERα to promote breast cancer cell proliferation and is frequently overexpressed in ERα positive breast cancer [5, 51]. Furthermore, overexpression of RET confers resistance to tamoxifen and aromatase inhibitors [5, 43]. RET mediated activation of the PI3K/ Akt and MAPK pathways leads indirectly to the phosphorylation and activation of ERα [5, 57]. These studies have identified RET as a potentially important target for the treatment of endocrine resistant breast cancer. A small molecule inhibitor with inhibitory activity towards RET, NVP-BBT594, has recently been shown to reverse resistance to aromatase inhibitors in breast cancer cells [43]. The effective use of kinase inhibitors to study the roles of RET in cell physiology will require the use of two or more structurally similar inhibitors [13, 21]. We have previously shown that SPP86 inhibits GDNF- RET induced activation of the MAPK pathway at low doses [45]. Herein, we have demonstrated that SPP86 inhibits GDNF- RET induced phosphorylation of ERα. Earlier studies have demonstrated that RET indirectly induces ERα phosphorylation via activation of the PI3K/Akt and MAPK pathways [5, 57]. Interestingly, SPP86 appeared more effective at inhibiting the GDNF- RET induced activation of the PI3K/Akt pathway than the MAPK pathway (0.1 vs 1.0 μM). Furthermore, SPP86 inhibited GDNF- RET induced ERα phosphorylation at concentrations similar to those required to inhibit Akt phosphorylation. This is in agreement with previous findings showing that inhibition of PI3K/Akt signaling is more effective at blocking ERα phosphorylation that inhibition of the MAPK pathway [5]. It is possible however, that SPP86 mediated inhibition of Src family kinases enhances its effect on Akt phosphorylation [45, 58]. ERα induces RET expression which in turn enhances ERα phosphorylation and activation in a positive feedback loop [5, 6, 51]. In our study, SPP86 inhibited the proliferation of MCF7 cells cultured in the presence of estrogen and GDNF to the same degree as tamoxifen on a molar basis. In contrast, SPP86 did not inhibit the proliferation of MCF7 cells cultured in the presence of estrogen and insulin. Exposure to SPP86 was also associated with a reduction in cyclin D1 levels. Cyclin D1 is a transcriptional target of ERα and central regulator of cell cycle progression in MCF7 cells [59, 60]. SPP86 thus appears to suppress MCF7 proliferation at least in part, by inhibiting ERα- RET cross talk and cyclin D1 expression.

Both sorafenib and SPP86 inhibited PI3K/Akt and MAPK pathway signaling to similar degrees. Our studies thus show that SPP86 selectively inhibits RET- induced MCF7 cell proliferation. Additional targets of individual kinase inhibitors with activity towards RET include the Aurora kinases, BRAF, EGFR, JAK2, KIT, MET, p38, PDGFRα/β and Src (Table 1). As these kinases all play roles in regulating MCF7 proliferation and/or survival, these inhibitors cannot be used in isolation to determine the cellular functions of RET. Although SPP86 shows inhibitory activity towards Src family kinases, it does not inhibit the aforementioned kinases. Additional targets of SPP86 such as EPHA1 and FLT4 (VEGFR3) play minor roles in regulating MCF7 proliferation and survival [61, 62]. Its selectivity and differential target profile make SPP86 an additional useful inhibitor for studies on RET function in human breast cancer cell lines.

## Conclusions

We have demonstrated that SPP86, a novel kinase inhibitor, is a useful tool for studying the cellular functions of RET. Numerous studies have identified RET as a potentially important therapeutic target in subtypes of breast, lung and thyroid cancers. Kinase inhibitors are useful tools for studying the cellular functions of kinases. Their relative lack of specificity can however, lead to erroneous results. The use of two or more structurally distinct kinase inhibitors has therefore been recommended for studies on cell physiology. Our studies have identified SPP86 as a selective inhibitor of RET signaling in human cancer cell lines. The selectivity profile of SPP86 is similar to that of PP1 and PP2 but differs substantially from that of other inhibitors that target RET. Unlike PP1 and PP2 however, SPP86 does not inhibit p38, CSK, KIT, PDGF, Src or BCR-ABL. Together, our findings indicate that SPP86 is a useful tool for studying the cellular functions of RET.

## Electronic supplementary material

Additional file 1: Figure S1: Inhibition of RET phosphorylation. **(A)** MCF7 cells were grown in media growing 1.0% FBS overnight, pretreated with 2.5 μM PF573228 and/or 1 μM SPP86 for 40 min and then exposed to 10 ng/ml of GDNF for a further 20 min in similar media. Total lysates were resolved by SDS-PAGE and probed with antibodies directed against phosphorylated and total Akt. Actin was used as a loading control. (PPTX 194 KB)
