# Supplementary figures and images for: Selective inhibition of RET mediated cell proliferation in vitro by the kinase inhibitor SPP86
Source: BMC Cancer. 2014 Nov 20;14:853. doi: 10.1186/1471-2407-14-853 (PMC4252022; doi:10.1186/1471-2407-14-853)

## Slide 1
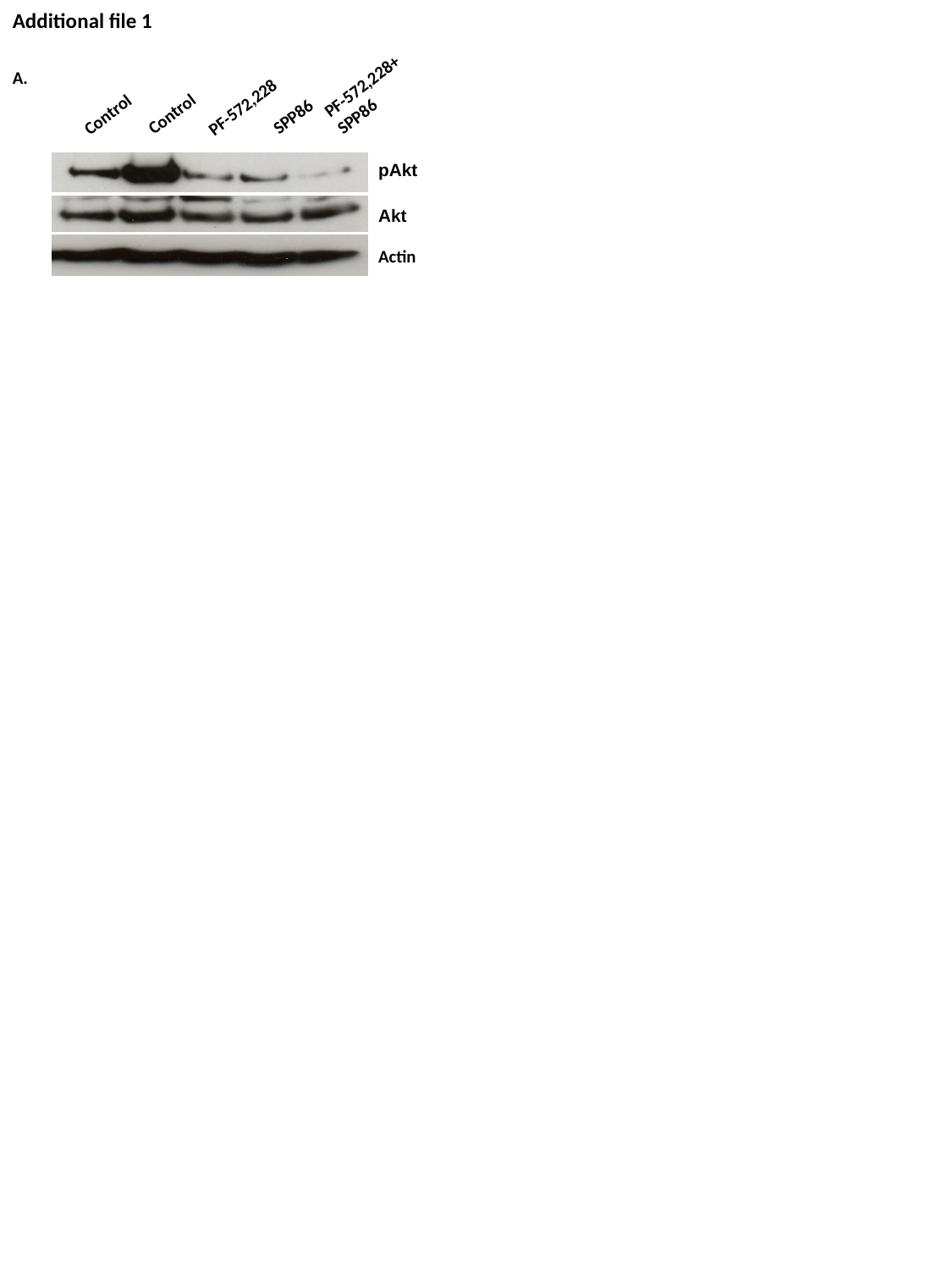

Additional file 1
A.
PF-572,228+ SPP86
PF-572,228
Control
SPP86
Control
pAkt
Akt
Actin

Supplement: Supplementary file 1 — Additional file 1: Figure S1: Inhibition of RET phosphorylation. (A) MCF7 cells were grown in media growing 1.0% FBS overnight, pretreated with 2.5 μM PF573228 and/or 1 μM SPP86 for 40 min and then exposed to 10 ng/ml of GDNF for a further 20 min in similar media. Total lysates were resolved by SDS-PAGE and probed with antibodies directed against phosphorylated and total Akt. Actin was used as a loading control. (PPTX 194 KB) [file 12885_2014_5047_MOESM1_ESM.pptx]
